# Nucleo-cytoplasmic shuttling dynamics of the transcriptional regulators XYR1 and CRE1 under conditions of cellulase and xylanase gene expression in *Trichoderma reesei*

**DOI:** 10.1111/mmi.12824

**Published:** 2014-10-29

**Authors:** Alexander Lichius, Verena Seidl-Seiboth, Bernhard Seiboth, Christian P Kubicek

**Affiliations:** 1 Research Division Biotechnology and Microbiology, Institute of Chemical Engineering, Vienna University of TechnologyVienna, Austria; 2 Austrian Center of Industrial BiotechnologyGraz, Austria

## Abstract

*Trichoderma reesei* is a model for investigating the regulation of (hemi-)cellulase gene expression. Cellulases are formed adaptively, and the transcriptional activator XYR1 and the carbon catabolite repressor CRE1 are main regulators of their expression. We quantified the nucleo-cytoplasmic shuttling dynamics of GFP-fusion proteins of both transcription factors under cellulase and xylanase inducing conditions, and correlated their nuclear presence/absence with transcriptional changes. We also compared their subcellular localization in conidial germlings and mature hyphae. We show that cellulase gene expression requires *de novo* biosynthesis of XYR1 and its simultaneous nuclear import, whereas carbon catabolite repression is regulated through preformed CRE1 imported from the cytoplasmic pool. Termination of induction immediately stopped cellulase gene transcription and was accompanied by rapid nuclear degradation of XYR1. In contrast, nuclear CRE1 rapidly decreased upon glucose depletion, and became recycled into the cytoplasm. In mature hyphae, nuclei containing activated XYR1 were concentrated in the colony center, indicating that this is the main region of XYR1 synthesis and cellulase transcription. CRE1 was found to be evenly distributed throughout the entire mycelium. Taken together, our data revealed novel aspects of the dynamic shuttling and spatial bias of the major regulator of (hemi-)cellulase gene expression, XYR1, in *T. reesei*.

## Introduction

In nature fungi contribute as essential decomposers of complex organic molecules to the biological carbon cycle. These organic polymers, produced through carbon dioxide fixation by plants, mainly comprise plant cell wall polysaccharides including cellulose, hemicelluloses, pectins and the polymer lignin. The major plant cell wall component is the β-(1,4)-linked glucose polymer cellulose, that alone exhibits an annual production of 7.2 × 10^10^ tons, and its microbial degradation is therefore a key transformation step in the biological carbon cycle. The ascomycete *Trichoderma reesei* (the anamorph of the pantropical saprotroph *Hypocrea jecorina*) has become a model system for the regulation of formation of plant cell wall polysaccharide hydrolases such as cellulases and hemicellulases (Seiboth., 2012; Kubicek, 2013). Notably, its hyperproducing strains are applied for the industrial production of the respective (hemi)cellulase enzymes for their applications in the pulp and paper, food and textile industry, as well as for the conversion of plant biomass materials into second-generation biofuels or platform chemicals (Chundawat., 2011; Viikari., 2012).

Most *T. reesei* cellulases are not formed during growth on monosaccharides and their full expression requires the presence of an inducer. Cellulase gene expression is regulated by the action of at least four positive transcriptional activators, including the xylanase regulator 1 (XYR1), the activators of cellulase 2 (ACE2) and 3 (ACE3), and a tetramer of heme-activated proteins (HAP2/3/5 complex), as well as two repressors, including ACE1 and the carbon catabolite repressor CRE1 (Kubicek., 2009; Seiboth., 2012; Häkkinen., 2014).

XYR1 is a Zn_2_Cys_6_-type regulator protein that binds to a 5′-GGCW_4_-3′ DNA motif and is the key transcriptional activator of cellulase and xylanase gene expression in *T. reesei*. Cellulases are coordinately upregulated through XYR1 activity upon induction by cellulose, lactose, or sophorose (Foreman., 2003; Kubicek., 2009), whereas expression of xylanases is differentially regulated. Expression of *xyn1* and *xyn2*, for instance, is induced by d-xylose (Herold., 2013), whereas expression of *xyn2* is also upregulated in the presence of the cellulase inducing carbohydrates cellulose and sophorose (Zeilinger., 1996). Furthermore, *xyn1* and *xyn2* respond to carbon catabolite repression in different ways (Mach., 1996). Deletion of *xyr1* completely eliminates the induction of both cellulases and xylanases by all known inducers (i.e. cellulose, lactose, sophorose, xylan, xylose and arabinose) and in addition XYR1 also regulates lactose, l-arabinose and d-xylose metabolism mainly at the level of the d-xylose reductase (Stricker., 2006; Seiboth., 2007; Akel., 2009). Similar regulatory dynamics of XYR1 or its XlnR orthologs occur in other species, including *Aspergillus* spp., *Neurospora crassa*, and *Fusarium* spp., although species-specific adaptations have been found (van Peij., 1998; de Vries., 1999; Hasper., 2000; Marui., 2002; Calero-Nieto., 2007; Stricker., 2008; Seiboth., 2012; Sun., 2012).

*Xyr1* transcription under non-inducing conditions occurs at a low basal level. It appears not to be further induced during growth on xylan (Mach-Aigner., 2008), but to be clearly induced by lactose and cellulose (Portnoy., 2011b; Bischof., 2013). So far, no information is available on how XYR1 reaches the nucleus and becomes active for cellulase transcription. Nevertheless, the majority of Zn_2_Cys_6_ regulators are permanently present in the nucleus (MacPherson., 2006), and only a minority – like *Aspergillus* AflR, NirA and AmyR – have so far been demonstrated to be shuttled between the cytoplasm and the nucleus (Shimizu., 2003; Berger., 2006; Makita., 2009).

Carbon catabolite repression by CRE1 (or its CreA orthologs in other filamentous fungal species), a member of the C_2_H_2_ class of DNA-binding proteins that recognizes a 5′SYGGRG-3′ consensus sequence (Felenbok., 2001), is the major negative regulator of cellulase and hemicellulase gene expression because it inhibits both basal and the inducible expression, and moreover also prevents expression of the positive regulator gene *xyr1* (Mach-Aigner., 2008; Seiboth., 2012). Carbon catabolite repression by CRE1/CreA appears to occur in different ways in different fungi: in *T. reesei* and *Aspergillus nidulans*, the *cre1*/*creA* genes are auto-regulated and transcribed at lower levels in repressing conditions (Arst., 1990; Strauss., 1995; Ilmen., 1996; Shroff., 1996), whereas glucose-dependent transcriptional expression patterns of *cre1* are positively correlated with the extracellular glucose concentration in *Acremonium chrysogenum* (Jekosch and Kück, 2000). Further, nuclear localization of CRE1 in *Sclerotium sclerotiorum* is dependent on the presence of glucose (Vautard-Mey., 1999), whereas nuclear localization of CreA is independent of glucose in *A. nidulans* (Roy., 2008).

Up to now, the shuttling of positive and negative transcription factors during cellulase and hemicellulase gene expression is completely obscure. The objective of this article therefore was to set up a system that allows the quantification of subcellular localization of XYR1 and CRE1 in *T. reesei* under cellulase and xylanase inducing and non-inducing conditions, and thus to provide the first temporal resolution of transcription factor shuttling during the induction of cellulase gene expression in *T. reesei*. The study reveals some interesting differences between the subcellular localization of XYR1 and CRE1 in germlings growing in submerged cultures and the functionally stratified, mature colony as it develops on agar plates under laboratory conditions. Our data show that cellulase gene expression depends on *de novo* biosynthesis and immediate nuclear import of XYR1, and furthermore suggest that XYR1-mediated cellulase expression occurs mainly in the central area of the colony. This provides the first evidence that the mycelial organization of the fungal colony might be another important element that regulates the high-level production of cellulolytic enzymes.

## Results

### XYR1 requires N-terminal and CRE1 requires C-terminal GFP labeling

In order to test potential influences of GFP labeling on the function of the two transcription factors, we first compared the basic subcellular localization patterns of N- and C-terminally labeled XYR1 and CRE1 fusion proteins, expressed under native conditions from their endogenous loci (Fig.S1). Although all four versions, i.e. XYR1–GFP, GFP–XYR1, CRE1–GFP and GFP–CRE1, became recruited into nuclei under inducing or repressing conditions, respectively, we also noted significant differences in their individual expression level and efficiency of nuclear recruitment, judged by localized fluorescence intensity measurements: attachment of GFP to the C-terminus of XYR1 resulted in approximately eightfold reduced expression and nuclear recruitment compared to the GFP–XYR1 version, whereas opposite effects, i.e. about fivefold reduced expression and nuclear recruitment of the N-terminal GFP version, were observed for CRE1 (Fig.S1).

To verify that the fusion proteins remained functional, *Δxyr1* and *Δcre1* gene deletion strains of *T. reesei* were transformed with each of the four fusion constructs: thereby expression of GFP–XYR1 completely rescued the growth defect of Δ*xyr1* on 1% (w/v) xylose medium, whereas XYR1–GFP did so only partially (Fig.S2). Likewise, the severe growth defect of Δ*cre1* was fully rescued by expression of CRE1–GFP, whereas strains complemented with GFP–CRE1 retained significantly reduced colony extension rates (Fig.S2). These data strongly suggested that XYR1 requires N-terminal and CRE1 requires C-terminal GFP labeling in order to preserve protein function. Consequently, only *T. reesei* transformants expressing GFP–XYR1 and CRE1–GFP, respectively, were used for all subsequent experiments.

### The subcellular localization dynamics of GFP–XYR1 and CRE1–GFP reporter proteins reflect the metabolic impact of the utilization of simple and complex carbon sources

**Figure 1:**
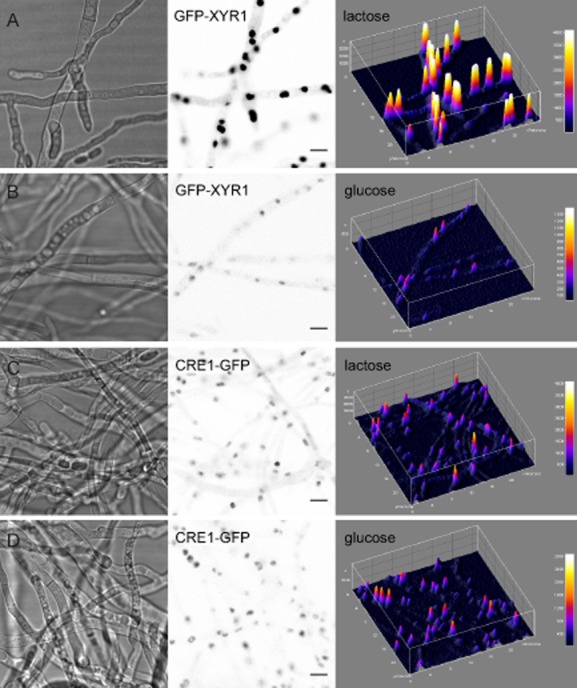
Nuclear import of XYR1 was significantly increased under cellulase inducing conditions.A and B. (A) GFP–XYR1 became strongly recruited into nuclei in the presence of lactose, whereas it was only weakly expressed under repressing/non-inducing conditions on glucose (B).C and D. CRE1 did not show this specific response and appeared equally recruited to nuclei under cellulase inducing and repressing conditions. 3D surface plots (right row) of the inverted fluorescence images (middle row) illustrate the relative differences of localized fluorescence signal intensities. The corresponding transmission light images are shown in the left row. The displayed strains were incubated for 48 h at 28°C on MA agar plates supplemented with lactose or glucose respectively.Scale bars, 5 μm.

Initial tests showed that CRE1 appeared to be equally localized to nuclei under cellulase inducing (lactose) and repressing (glucose) conditions, whereas XYR1 showed a significant increase of nuclear import only under cellulase inducing conditions (Fig. 1). The distinct shuttling responses between both fluorescently labeled transcription factors, and the apparently specific, i.e. induction-dependent, response of GFP–XYR1 strongly suggested that both fusion proteins can be used as diagnostic tool to monitor cellulase gene expression in *T. reesei*.

This notion was further substantiated by comparing the subcellular localization dynamics of GFP–XYR1 and CRE1–GFP after replacement onto various other cellulase inducing and non-inducing carbon sources (Fig.S3). The most potent carbon source triggering nuclear import of XYR1 was sophorose, followed by lactose and cellulose, which both required extended incubation times to lead to a noticeable increase in nuclear fluorescence compared to the pre-culture. For CRE1, the most potent carbon sources triggering nuclear import were found to be glucose, high concentrations (65 mM) of xylose, and glycerol. Together these data demonstrate that on simple, easily metabolized carbon sources, nuclear accumulation of both transcription factors reached high levels within 2–4 h after carbon source replacement, whereas on more slowly metabolized, complex carbon sources more than 18 h were required to reach similar levels.

**Figure 2:**
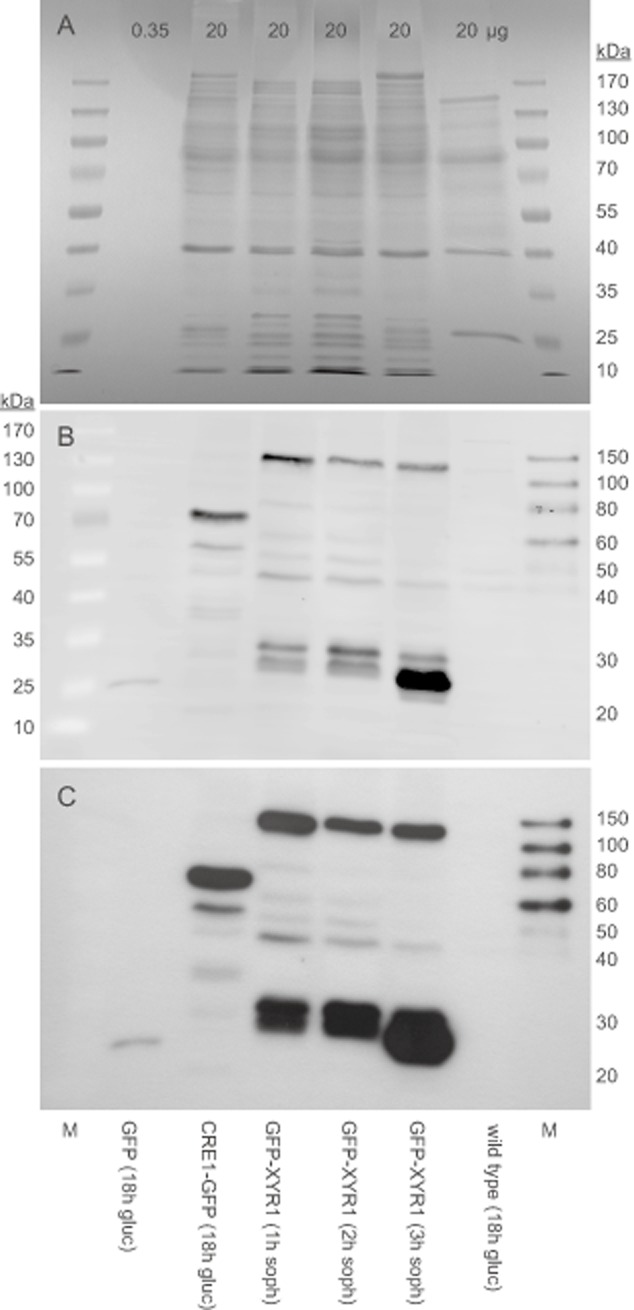
Protein turn-over kinetics of both fusion reporters differ, leading to significant nuclear accumulation of free GFP from GFP–XYR1 degradation.A. Coomassie-stained SDS-PAGE gel. Except for the GFP positive control, of which only 0.35 μg total protein extract of QM6a P*_tef1_*::*gfp* were loaded, 20 μg of all other protein extract samples were loaded per lane. Molecular weight marker on both sides is PageRuler 10–170 kDa ladder.B. Western blot of the sister SDS-PAGE gel shown in (A), incubated with monoclonal α-GFP(B-2) antibody, ECL 2 reagent and imaged on a Typhoon FLA700 chemifluorescence imager. Molecular weight marker on the left (lane 1) is PageRuler 10–170 kDa ladder, on the right (lane 8) is SuperSignal Enhanced 20 – 150 kDa ladder. Cytosolic GFP (26.98 kDa) as positive control is exclusively detected in lane 2. Full-length CRE1–GFP (71.07 kDa) bands in lane 3 just above the 70 kDa marker, and some of its degradation products with decreasing abundance towards low-molecular-weight species below. Lanes 4–6 show full-length GFP–XYR1 (128.41 kDa) with decreasing abundance after 1–3 h of sophorose induction. Interestingly, with time, abundance of its higher-molecular-weight degradation products (40–80 kDa) decreases, while amounts of its low-molecular-weight degradation products (25 – 30 kDa) significantly increase. The negative control in lane 7 (protein extract from an 18 h glucose culture of QM9414 Δt*ku70*) reveals minor non-specific background detection in the higher molecular weight range > 40 kDa.C. The same membrane as shown in (B) developed by x-ray film exposure, shows identical results with less non-specific background signals, however, inferior spatial resolution. Intense nuclear fluorescence of GFP–XYR1 and CRE1–GFP, respectively, was confirmed by live-cell imaging in all samples prior to biomass harvest and protein extraction.

In order to confirm that GFP–XYR1 and CRE1–GFP were expressed as full-length proteins and hence responsible for the observed changes in subcellular fluorescence, SDS-PAGE and Western blot analyses were performed (Fig. 2). Full-length CRE1–GFP (71.07 kDa) was readily detected from crude protein extracts of TRAL003.1 after 18 h growth on glucose medium. Several degradation products, most prominently banding at around 60 and just below 40 kDa, were also detected in minor amounts. Full-length GFP–XYR1 (128.41 kDa) was also readily detected in protein extracts of TRAL002.1 induced by sophorose for 1, 2 and 3 h. However, in this case a higher concentration of apparent degradation products of GFP–XYR1, most noticeably banding at around 25–30 kDa, were already seen at the earliest time point and increased throughout the time period, suggesting a higher proteolytic susceptibility of the GFP–XYR1 construct. The very prominent degradation product of about 25 kDa after 3 h is most likely the mere GFP (Fig. 2B and C), indicating that GFP–XYR1 degradation leaves GFP intact.

In order to rule out that free GFP, resulting from hypothetical GFP–XYR1 or CRE1–GFP degradation in the cytoplasm, enters the nucleus and causes the observed increase in nuclear fluorescence, we used a control strain that expresses GFP under the constitutive P*tef1* promoter. Nuclei in that strain stained negatively against the brightly fluorescent cytoplasm, strongly suggesting that GFP does not readily cross the nuclear envelope (Fig.S4). Hence, the observed changes in nuclear fluorescence must be due to shuttling of the full-length GFP-transcription factor fusion proteins.

### XYR1 generally shuttles slower than CRE1 and it is not maintained as cytoplasmic pool

**Figure 3:**
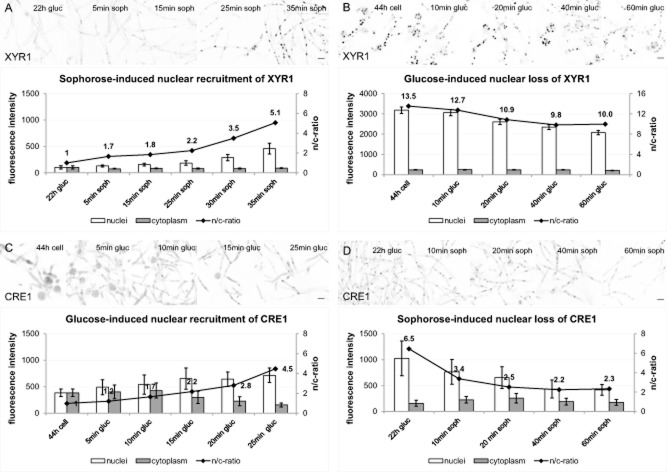
XYR1 and CRE1 showed distinct nuclear shuttling dynamics. Inverted fluorescence images show the degree of nuclear transcription factor recruitment at representative time points, with the corresponding quantitative analysis below.A. Time-course of sophorose-induced nuclear import of GFP–XYR1. Quantitative image analysis showing immediate but weak nuclear recruitment which started to significantly increases after 30 min upon induction, probably correlated with the maturation time of newly synthetized GFP.B. Time-course of glucose-induced nuclear loss of GFP–XYR1. Quantitative image analysis showing only slow decrease of nuclear XYR1 signal within the first hour upon carbon source replacement.C. Time-course of glucose-induced nuclear import of CRE1–GFP. Quantitative image analysis showing rapid nuclear import of CRE1 from the cytoplasmic pool.D. Time-course of sophorose-induced nuclear loss of CRE1–GFP. Quantitative image analysis showing rapid nuclear export of CRE1 upon cellulase induction with sophorose. Please refer to the text for more detailed explanation.Scale bars, 5 μm.

We next investigated the time needed for XYR1 and CRE1 to shuttle between the cytoplasm and the nucleus. To this end, we monitored subcellular dynamics of both transcription factors in conidial germlings in real-time upon replacement onto a new carbon source (Fig. 3). Nuclear recruitment of XYR1 and nuclear loss of CRE1, respectively, were triggered by addition of sophorose to germlings pre-cultured for 22 h in glucose, whereas nuclear loss of XYR1 and nuclear recruitment of CRE1, respectively, were initiated by addition of glucose to germlings pre-cultured for 44 h on cellulose.

Nuclear recruitment of XYR1 was detectable within 5 min after addition of sophorose, however, required about 30 min more until a significantly high increase in nuclear signal (n/c-ratio tripled) could be observed (Fig. 3A). Interestingly, cytoplasmic fluorescence intensity remained constant over the whole induction period, while the nuclear signal increased steadily. The fact that the cytoplasmic signal of XYR1 remained constantly low during that period indicates that any newly produced XYR1 became immediately imported into nuclei. On the other hand, nuclear loss of XYR1, triggered by transfer to glucose-containing medium, occurred very slowly and about 65% of nuclear fluorescence was still visible after 60 min (Fig. 3B). The cytoplasmic pool of XYR1 did not increase during this period, suggesting either that significant export of GFP–XYR1 from the nucleus did not occur, or that GFP, as stable end-product of GFP–XYR1 degradation, remained inside the nucleus. In any case, these data indicate that XYR1 is only produced when needed, i.e. in response to a cellulase inducing signal, and that a significant cytoplasmic pool of the transcription factor is not maintained.

For CRE1, the nucleo-cytoplasmic shuttling dynamics were strikingly different. Already after 5 min upon replacement onto glucose, clear nuclear recruitment could be observed, and it took only another 15 min to triple the n/c-ratio (Fig. 3C). However, comparing the fluorescence intensity in the nucleus and in the cytoplasm after 25 min revealed that there was only a small increase (1.6-fold increase) in total CRE1, and the 4.5-fold n/c-ratio increase was due to a reduction of the cytoplasmic concentration. This indicates that nuclear import of CRE1 occurred almost instantly (within less than 5 min) and exclusively from a pre-formed cytoplasmic pool. An equally fast response was observed for sophorose-induced loss of nuclear CRE1. In this scenario, the nuclear signal of CRE1 dropped very rapidly (by a factor of 1.9 within 10 min), coinciding with an initial increase in the cytoplasmic signal, which subsequently, however, stabilized at a medium level (Fig. 3D).

### XYR1 nuclear recruitment dynamics correlate with rates of cellulase and xylanase gene expression

**Figure 4:**
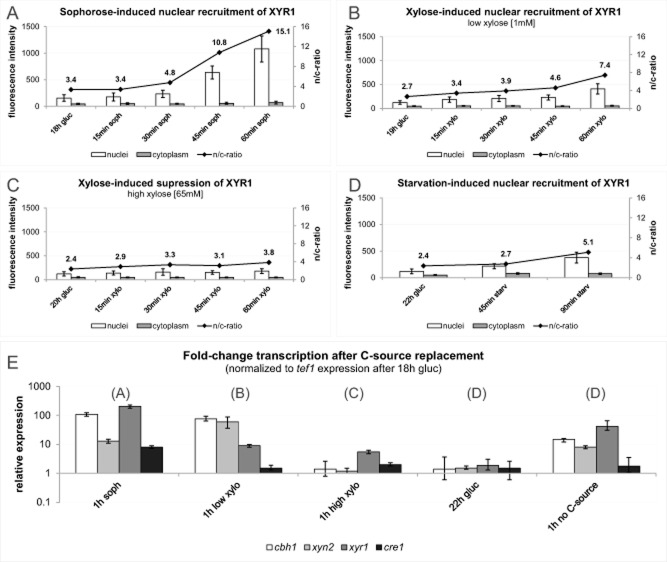
XYR1 nuclear import dynamics correlated with gene expression.A. Sophorose-induced nuclear import of XYR1 represents cellulase inducing conditions.B. Low-xylose (1 mM) induced nuclear import of XYR1 represents cellulase and xylanase inducing conditions.C. High-xylose (65 mM) induced XYR1 suppression represents carbon-catabolite repressing conditions.D. Starvation-induced nuclear import of XYR1 represents carbon-catabolite de-repressing conditions.E. Gene expression analysis from biomass samples collected at the end of each imaging experiment quantified in (A–D) showed that upon sophorose addition cellulase *cbh1* and *xyr1* itself were strongly upregulated, low concentration (1 mM) of xylose strongly induce *cbh1* and *xyn2*, but not *xyr1*, whereas in the presence of 65 mM xylose none of these three marker genes was significantly upregulated. This is different under de-repressing conditions, i.e. in the absence of any experimentally added carbon source, where nuclear import of XYR1 evidently induced moderate upregulation of *cbh1*, *xyn2* and of itself.

Since the nuclear presence of a transcription factor does not necessarily imply that it is also transcriptionally active, we tested whether carbon source-induced nuclear import of XYR1 would correlate with cellulase and xylanase gene expression. As shown in Fig. 4, using sophorose as an inducer, the nuclear presence of XYR1 correlated with upregulation of the major cellulase gene (*cel6a*/*cbh1*), as well as its own (*xyr1*) upregulation, whereas the expression of the major xylanase gene (*xyn2*) was only marginally increased. When low concentrations (1 mM) of xylose were used for induction, the comparably moderate and slower increase in nuclear XYR1 signal correlated with *cbh1* transcription and significantly higher *xyn2* expression compared to sophorose. Neither cellulase nor xylanase gene expression were detected at high concentrations (65 mM) of xylose or on glucose. Interestingly, under carbon starvation (= incubation without any carbon source), moderate *cbh1* gene expression was detected, which coincided with weak *xyr1* expression and only slightly elevated nuclear recruitment of XYR1. *Cre1* transcript levels did not show any significant regulation under these conditions.

In order to rule out that mechanical stress during biomass transfer for carbon source replacement disturbed the cells and introduced artificial responses, we performed control experiments in which the inductive sophorose pulse was directly administered into the glucose pre-culture medium. No significant changes in GFP–XYR1 import dynamics nor subsequent marker gene expression could be observed (Fig.S5). Initiation of GFP–XYR1 *de novo* production, its nuclear import and the subsequent expression of cellulase and hemicellulase genes occurred equally well in the presence of sophorose and residual amounts of glucose as they did in the presence of sophorose alone. This confirmed that the applied carbon source replacement procedure did not introduce mechanical stress into the assay. It also showed that residual amounts of glucose did not interfere with sophorose-mediated cellulase induction.

### XYR1-mediated gene regulation depends on its own *de novo* protein biosynthesis

**Figure 5:**
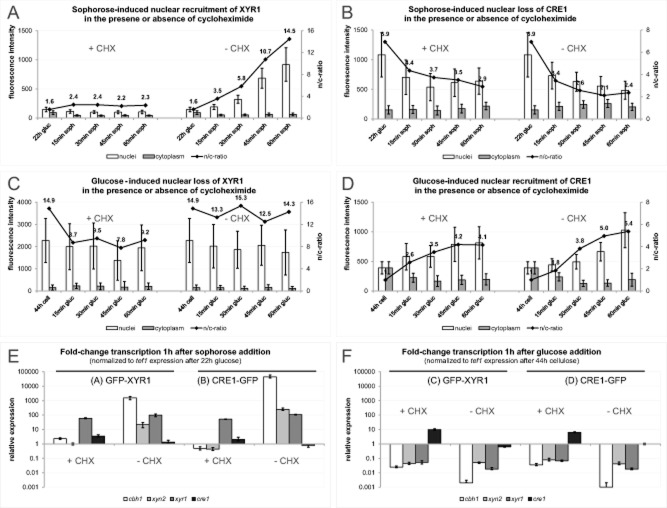
Complete inhibition of XYR1 nuclear import and cellulase gene expression by cycloheximide treatment. (A)–(D) show the shuttling dynamics of fluorescently labeled XYR1 and CRE1 upon cellulase induction and repression, in the presence and absence of 50 μM cycloheximide (CHX) respectively. (E) and (F) show the corresponding transcriptional analyses.A. Addition of CHX completely blocked *de novo* protein biosynthesis of XYR1 and consequently its nuclear import.B. Nuclear loss of CRE1 remained unaffected under the same conditions.C. Nuclear loss of XYR1, on the other hand, was slightly enhanced in the presence of CHX in comparison to the untreated control.D. Glucose-induced nuclear import of CRE1 showed no significant differences between CHX-treated sample and untreated control.E. Inhibition of *de novo* XYR1 protein biosynthesis and nuclear import was directly correlated to the inhibition of transcription of XYR1-dependent genes (*cbh1* and *xyn2*). Transcription of *xyr1* itself, however, responded normally to the induction signal and became upregulated to the same extend as in the untreated controls.F. Apart from a weak upregulation of *cre1* in the presence of CHX, all other tested marker genes became downregulated as expected under cellulase non-inducing conditions.

The slightly delayed nuclear import of XYR1 in comparison to CRE1, and its own rapid upregulation under cellulase inducing conditions suggested that *de novo* protein biosynthesis of XYR1 is required for its gene regulatory function. To test this hypothesis we compared transcription factor shuttling dynamics and marker gene expression in the presence and absence of the protein biosynthesis inhibitor cycloheximide (CHX) (Schneider-Poetsch., 2010). The results clearly show that in the presence of 50 μM CHX, biosynthesis and intense nuclear accumulation of XYR1 were completely blocked (Fig. 5A), and consequently XYR1-dependent genes (*cbh1* and *xyn2*) were not upregulated (Fig. 5E). *Xyr1* gene expression, however, remained largely unaffected by CHX treatment. In the presence of CHX, a slightly enhanced nuclear loss of XYR1 was observed (Fig. 5C), whereas no significant effects of the inhibitor on the shuttling dynamics and transcriptional activity of CRE1 were found (Fig. 5B, D and F). The latter confirming that nuclear import and transcriptional activity of CRE1 did not depend on its own *de novo* biosynthesis, at least not during the initial hour following carbon source replacement.

### Import of XYR1 is elevated in nuclei of the central areas of the mycelium

The nature of the available carbon source has a strong impact on colony development, particularly the rate of colony extension, hyphal density and onset and degree of conidiation (Fig.S2). In general, cellulase non-inducing, carbon catabolite repressing conditions [glucose or high concentrations (65 mM) of xylose] promote faster growth and an increased rate of hyphal branching while delaying conidiation, whereas cellulase inducing conditions (sophorose or cellulose) result in slower and sparser growing hyphae, and an early onset of conidiation. Lactose results in an intermediate phenotype with respect to these morphological features.

**Figure 6:**
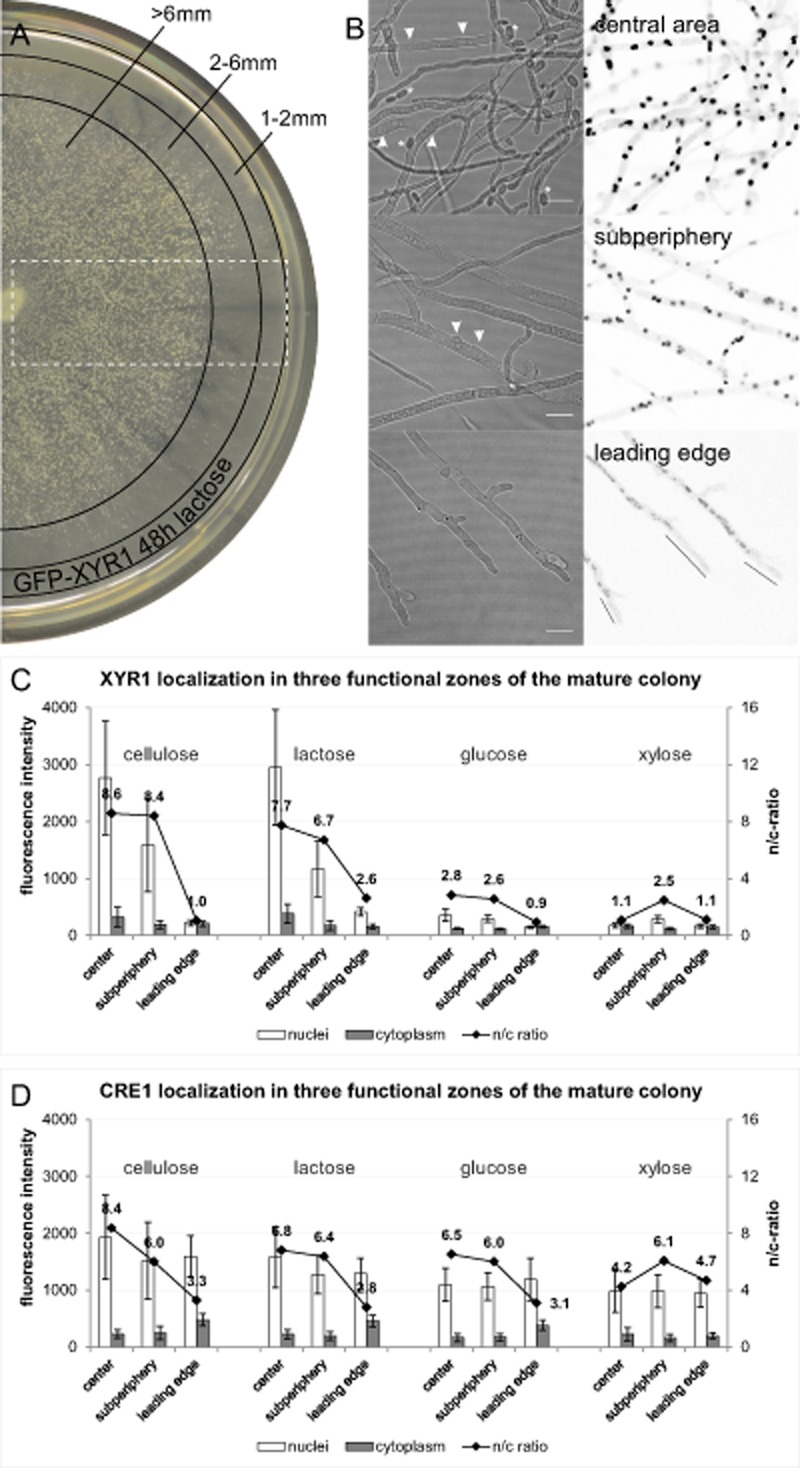
Transcription factor recruitment in the functionally stratified colony.A. Forty-eight-hour lactose plate culture of a GFP–XYR1 expressing strain. The white dotted rectangle indicates the sampling area which includes all three main functional zones of the colony (central area, subperiphery and leading edge) assessed by live-cell imaging microscopy.B. Bright-field images show the typical hyphal phenotypes: dense hyphal ‘network’ with conidiation (conidia indicated with asterisks) and a high degree of vacuolation (vacuoles indicated with arrowheads) in the central area, branched hyphae with beginning vacuolation but no conidiation in the subperiphery, and largely non-vacuolated leading hyphae with clear apical nuclear-exclusion zone (indicated with black lines). Representative images of the GFP–XYR1 fluorescence signal from these three areas show the evident decrease in nuclear XYR1 signal from colony center to colony edge. Scale bars, 5 μm.C and D. Quantification of nuclear recruitment of XYR1 and CRE1, respectively, in these three zones on inducing (cellulose and lactose) and non-inducing/repressing (glucose and xylose) carbon sources showed that nuclear import of XYR1 peaked in the central colony area exclusively under cellulase inducing conditions. CRE1, on the other hand, was equally recruited to nuclei in all regions of the colony with no significant differences between the tested carbon sources. All cultures have been incubated for 48 h at 28°C on MA medium supplemented with 1% w/v of the respective carbon source.

In order to assess how XYR1 and CRE1 nuclear recruitment are correlated with colony development, we analyzed the subcellular localization dynamics of both transcription factors in three distinct zones of the functionally stratified mycelium: the leading edge of the colony periphery, the subperiphery and the central area (Fig. 6).

XYR1 showed a steep distribution gradient across the colony profile on cellulose and lactose, with its strongest nuclear localization occurring in the central colony area. Its nuclear concentration in leading hyphae at the colony periphery was comparable to that in the cytoplasm. Similar data were also obtained with sophorose induction, although under these conditions also the cytoplasmic pool of XYR1 was increased in the central area, resulting in a lower n/c-ratio than in the subperiphery, although overall nuclear accumulation was considerably higher (Fig.S6). This may indicate that the biosynthesis of XYR1 occurs predominantly in the central area, followed by the subperiphery. CRE1, on the other hand, was found to be strongly recruited to the nuclei in all regions of the colony, and this pattern was similar with any of the tested carbon sources.

## Discussion

The XYR1 cellulase regulator is a Zn_2_Cys_6_ binuclear zinc cluster protein (Stricker., 2006). It is commonly believed that most Zn_2_Cys_6_ zinc cluster proteins are localized within the nucleus on a constitutive and permanent basis (MacPherson., 2006). However, there is now an increasing number of these zinc cluster proteins from multicellular fungi known, which do not share this behavior (Shimizu., 2003; Berger., 2006; Makita., 2009; Dinamarco., 2012). Here we showed that the XYR1 transcription factor of *T. reesei* also joins this list. Interestingly, the majority of zinc cluster proteins permanently localized to nuclei, such as Lys14, Oaf1, War1, Put3, and Leu3, have been described in yeast and regulate amino acid or nucleotide biosynthesis (Axelrod., 1991; Kirkpatrick and Schimmel, 1995; El Alami., 2000; Kohlhaw, 2003; Kren., 2003; Braun., 2005). Of these, Oaf1, War1, Put3, and Leu3 are constitutively bound to their target promoters, and their transcriptional activity is controlled by direct interaction with the inducing metabolites (Sellick and Reece, 2003; 2005[Bibr b64],[Bibr b65]; Harbison., 2004). Evidently, this is a very efficient way to quickly induce transcription, especially in small cells in which the diffusion distance necessary for the metabolite to reach the binding protein is short. This mechanism, however, could be a significant shortcoming for filamentous fungi in which many processes depend on long-distance transport and sophisticated spatial regulation; especially in a multinucleate environment with non-synchronized nuclei. In addition, the localization of other yeast Zn_2_Cys_6_ regulators that are not involved in nitrogen metabolism – such as Gal4 (Wightman., 2008) – also depend on induction. We therefore believe that the generalization about nuclear localization of zinc cluster proteins may not be valid and/or only be true for such that are involved in processes critical for cell survival.

The way in which XYR1 receives the inductive signal is not yet clear. Nevertheless, here we have shown that upon cellulase induction, *de novo* protein biosynthesis of XYR1 is essential for it to enter the nucleus in sufficient amounts in order to upregulate cellulase and xylanase gene transcription, as well as its own expression. The fact that transcriptional activation of *xyr1* occurred also upon inhibition of *de novo* protein biosynthesis would argue against an auto-regulatory mechanism of *xyr1* transcription. However, pre-formed XYR1 present before the addition of cycloheximide might be sufficient to specifically trigger *xyr1* gene expression under these conditions. But because subsequent translation of *de novo xyr1* transcripts is blocked by the drug, XYR1 protein is missing to activate cellulase and xylanase gene expression.

The inducibility of XYR1 by cellulose or lactose has been demonstrated earlier (Portnoy., 2011b; Bischof., 2013), and is in line with the presented data. On xylose (1 or 65 mM) expression of *xyr1* was higher compared to glucose but still lower compared to the no carbon source condition. These findings raise the question as to how XYR1 receives its signal for activation of cellulase and xylanase gene expression. The correlation of nuclear recruitment of XYR1 and cellulase formation suggests that if activation would occur in the nucleus it must be very rapid. Alternatively, the same signal regulates its import and activation. However, the indicated auto-regulatory *de novo* biosynthesis mechanism of XYR1 following a cellulase inducing signal renders it also possible that no activation of XYR1 is in fact required, because it is only synthesized when needed. In *Aspergillus niger*, XlnR has been reported to be cytoplasmic in its inactive state and nuclear import of XlnR accompanies induction by 10 mM xylose (Hasper., 2004). Although this very likely reflects the general trend of xylose induced nuclear import of XlnR, which is consistent with XYR1 behavior under similar conditions (1 mM xylose) in *T. reesei*, overexpression of the GFP–XlnR construct under the constitutive *pkiA* promotor, however, might have distorted the amounts of the transcription factor found in the nuclei and cytoplasm under these conditions. Comparative data on XlnR subcellular localization dynamics under cellulase inducing or non-inducing conditions would indeed be very helpful at this point. A recent study identified large differences in the set of genes that are controlled by XlnR/XYR1 in five different filamentous fungal species, suggesting a fine-tuned (hemi-)cellulolytic regulatory system dependent on the occupied biotope (Klaubauf., 2014).

In contrast to XYR1, the cellular pool of CRE1 proved to be very stable and the addition of glucose or high xylose caused a very rapid shift from cytoplasmic to nuclear localization. This finding is consistent with an efficient carbon sensing mechanism that aids to almost instantly adjust *T. reesei* metabolism to changes in the nutrient status, in this case to the most favorable condition of available glucose. This behavior is essentially similar to that of the *Saccharomyces cerevisiae* carbon catabolite repressor Mig1 (De Vit., 1997).

The fast nuclear accumulation of both transcription factors was remarkable: increases in nuclear fluorescence of XYR1 and CRE1 could be measured within 5 min after carbon source replacement. In this context, it has to be taken into account that the maturation time of GFP delays detection of a newly synthesized fusion protein by about 30 min (Heim., 1995; Heim and Tsien, 1996), and thus these fast kinetics reflect the immediate import of preformed protein. It is conceivable that nuclear import of newly synthesized transcription factor occurs at the same rate, but due to the experimental limitation of GFP maturation is picked up with a delay. Possibly, with a more sensitive microscopy set up providing higher temporal resolution, and by using faster maturing and/or faster decaying GFP variants, such as TurboGFP (Evdokimov., 2006) or labile GFPs (Deichsel., 1999; Zhao., 1999), it could be demonstrated that the cells actually respond and reach higher nuclear transcription factor concentrations much quicker.

Another interesting aspect from this work was that the termination of transcriptional activity of CRE1 seemed to be accompanied by its nuclear export. At this time, we must emphasize that the mechanism involved has not been fully resolved yet, and we thus avoid the term ‘export’. Nevertheless, our data show that the decay of nuclear fluorescence upon carbon source replacement was very fast with CRE1 (50% reduction in about 10 min). As mentioned earlier, the observed transient increase in the cytoplasmic signal of CRE1 is probably caused through exported CRE1 that is used to replenish the cytoplasmic pool, at least to a certain extent. Cytoplasmic pools of CRE1 were generally significantly larger because CRE1 was not as rapidly degraded as XYR1, and furthermore, CRE1 shuttling and function was not affected by CHX treatment. Together these data suggest that, in contrast to XYR1, CRE1 is being recycled.

It is thus possible that CRE1 initially has to be exported from the nucleus to initiate its degradation, while this proteolysis later on also continues in the nucleus or acts immediately on the protein transported into the cytoplasm. Again, this is similar to *S. cerevisiae* Mig1 whose nuclear export signal is phosphorylated by Snf1 upon glucose removal, causing it to be recognized by the nuclear exportin Msn5 and carried out of the nucleus into the cytoplasm (DeVit and Johnston, 1999).

In contrast to CRE1–GFP, the fluorescence intensity of GFP–XYR1 in nuclei decreased only very slowly after termination of induction, and its cytoplasmic pool remained essentially unchanged. One explanation for this finding would be an inactivation of XYR1 in the nucleus by, for example, proteolysis. In fact, such nuclear degradation has been demonstrated for the model Zn_2_Cys_6_-type transcription factor Gal1 of *S. cerevisiae*, which is also degraded while still being bound to DNA (Muratani., 2005). The occurrence of such a mechanism for XYR1 would be in agreement with our findings that significant amounts of free GFP as products of GFP–XYR1 degradation remain inside the nucleus. Lacking an export signal, removal of free GFP from the nuclei is a slow process and explains the slow decay in fluorescence after termination of induction. The Western blot data revealed that the degradation of XYR1 is actually much faster.

Together with our observation that residual amounts of glucose do not interfere with the inductive carbon source, and thus do not terminate cellulase gene expression, this suggests a mechanism by which no terminating signal might be required to switch off cellulase and hemicellulase gene expression, but that solely depends on the presence of the inducer. The rapid destruction of nuclear XYR1 would ensure that shortly after the inductive carbon source has been used up and consequently XYR1 *de novo* synthesis stopped, the expression of XYR1-dependent genes would cease automatically. For Gal4 from *S. cerevisiae*, it has been demonstrated that two distinct modes of ubiquitin-mediated proteolysis (one independent and one dependent of transcription) are essential to restrict its function and to achieve productive activation respectively (Muratani., 2005; Collins and Tansey, 2006). This makes it plausible that a similar regulatory mechanism might operate to provide very tight control over XYR1 activity without the need of a terminating carbon source. As a result, cellulase expression only occurs as long as an inductive carbon source is present and terminates by itself when this signal is removed. From a cell biological point of view, it makes a lot of sense that a signal which is specifically and rapidly switched on upon perception of a cellulase inducing carbon source can be switched off equally fast. Our data strongly suggests that this off-switch involves rapid degradation of XYR1 inside the nucleus.

This model is further substantiated by the fact that rapid and extensive degradation was only observed for GFP–XYR1, but not CRE1–GFP, which suggests this phenomenon is specific to XYR1 and hence no artifact of the GFP fusion.

Cleavage of the GFP–XYR1 before nuclear import can also be excluded as this would have led to measurable increase in cytoplasmic fluorescence upon cellulase induction which was never observed.

Antibodies against XYR1 are unfortunately not available at the moment, but highly desirable tools to investigate further details of XYR1 function. To be useful in future studies, antibodies should be raised against an N-terminal epitope of the XYR1 protein. Otherwise, the chance of acquiring false negative results in Western blots is high in case a C-terminally located epitope has already been degraded. Detecting XYR1 as a GFP–XYR1 fusion construct with an anti-GFP antibody circumvented these shortcomings and allowed us to identify the various GFP–XYR1 degradation products in the first place.

The conditional loss of nuclear CRE1, on the other hand, raises the question as to the mechanism that triggers it: in the case of Mig1, a carbon catabolite repressor that shares high similarity with CRE1 in the DNA-binding region (Nehlin and Ronne, 1990), glucose regulates its localization by affecting its Snf1-kinase-dependent phosphorylation, causing it to be nuclear when glucose is present, and cytoplasmic when glucose is absent (DeVit and Johnston, 1999). However, this model is unlikely to be applicable to the fungal CRE1/CreA proteins, because they are not regulated by the Snf1 ortholog (Cziferszky., 2002; Roy., 2008), and mutations in putative phosphorylation sites do not alter the nuclear localization (Vautard-Mey., 1999). Furthermore, deletion of *snf1* in *Magnaporthe oryzae* and *Cochliobolus carbonum* did not lead to relief from carbon catabolite repression (Tonukari., 2000; Yi., 2008). The mechanism that leads to nuclear export and degradation of CRE1, therefore, remains to be determined.

Our data also demonstrated that XYR1 is not equally distributed in the hyphal nuclei, and in fact occurs mainly in nuclei of the central area of the colony. Genetic heterogeneity of the nuclei in hyphae has been observed also in other fungi (e.g. *Fusarium moniliforme*) and proposed to contribute to phenotypic plasticity and virulence (reviewed in Roper., 2011). Levin. (2007a), studying the protein expression profile of five different, arbitrarily selected zones of *A. niger* colonies, found that 9% of the genes were expressed in only one of the five concentric zones, illustrating that a considerable part of the genome is active in a restricted part of the mycelium only. These authors also reported that genes induced by XlnR in *A. niger*, were upregulated in the center of the colony (Levin., 2007a,b[Bibr b40],[Bibr b41]). Their findings are in accordance with the spatial accumulation of GFP–XYR1 containing nuclei in *T. reesei*, yet the reason for this is enigmatic. It is possible that these central hyphal compartments are already under carbon starvation, as reported for *A. niger* (Levin., 2007a), and therefore provide an undisturbed environment for induction. In this case, however, increased nuclear recruitment of XYR1 in this area would be expected to occur independently of the available carbon source and not specifically under cellulase inducing conditions. In germlings we saw a clear difference in the amount of nuclear XYR1 between cellulase inducing and de-repressing (starvation) conditions, thus it is conceivable that in the mature colony these two metabolic states are as well distinctly regulated. Alternatively, it could thus be possible that the inducer arises in the central compartment. Whether this implies that the central area is also the area of cellulase secretion is unclear: protein secretion in filamentous fungi is generally assumed to occur at growing hyphal tips, because only the growing cell wall would be porous enough to allow the secreted proteins to pass through. However, it has been shown in *A. niger* that α-amylase is secreted both at the hyphal tips and at the septa by exocytosis (Hayakawa., 2011; Read, 2011). No studies have so far been performed with *T. reesei*, but we assume in analogy that cellulases are also secreted at these two places, hence implying long-distance transport of their mRNA or the precursor proteins. What has been demonstrated recently, is a significant increase of the conidiation-associated transcriptome in *T. reesei*, including the cellulase and xylanase marker genes *cel6a/cbh1* and *xyn2* (Metz., 2011). As mentioned above, onset and degree of conidiation greatly depend on the available carbon source, and are usually restricted to the central colony area. This obviously provides a functional link between conidiogenesis and nuclear import of XYR1 in the same region. However, in the present study, we have restricted the analysis of transcription factor shuttling dynamics to vegetative hyphae of the mycelium, excluding aerial hyphae and conidiophores/conidia. In contrast, Metz. (2011) investigated transcriptional changes in a mixture of aerial hyphae, conidiophores and conidia. This leaves the interesting question as to how cellulase regulation and associated XYR1 and CRE1 transcription factor shuttling might differ between these distinct morphological stages, and certainly warrants further, more detailed investigation.

Taken together, the present data have revealed a dynamic shuttling and a spatial bias of the major regulator of cellulase and hemicellulase gene expression of *T. reesei*, XYR1, during the process of induction of expression of the respective hydrolase genes. This clearly offers new cellular areas for manipulation of *T. reesei* strains for cellulase production.

## Experimental procedures

### Strains and growth conditions

*Trichoderma reesei* strain QM9414 (ATCC 26921), an early cellulase overproducing mutant derived from the wild-type isolate QM6a (ATCC 26921) and the knock-out strains *Δxyr1* (Stricker., 2006), and Δ*cre1* (Portnoy., 2011a) were used throughout this work. *T. reesei* transformants expressing green fluorescent protein (GFP)-labeled fusion proteins were created in QM9414 or QM9414 Δ*tku70* (C. Ivanova., unpubl. data) parental strain backgrounds, in the latter the Δ*tku70* gene was replaced in strain QM9414 by the pyrithiamine marker (Kubodera., 2002) essentially as described (Guangtao., 2009), and ultimately comprised strains TRAL001 (*xyr1–gfp*), TRAL002 (*gfp–xyr1*), TRAL003 (*cre1–gfp*) and TRAL004 (*gfp–cre1*). QM9414 and QM9414 Δ*tku70* respectively, served as reference strains in all experiments. Strain propagation, transformant selection and purification were performed on potato dextrose agar (PDA, Difco). For experimentation, strains were grown in Mandels–Andreotti (MA) medium (Mandels and Andreotti, 1978), using glucose, xylose, glycerol, lactose, sophorose or cellulose as sole carbon sources at final concentrations as indicated below. All strains are maintained as glycerol stocks at −80°C. All strains used and generated in this study are listed in Table S1.

*Escherichia coli* strains JM109 (cat.no. P9751, Promega, Madison, Wisconsin), One Shot®Top10 (cat.no. C4040-10, Life Technologies-Invitrogen, Austria) or Stellar® (cat.no. 636763,Takara Bio Europe/Clontech, Saint-Germain-en-Laye, France) were used for plasmid construction and amplification using standard molecular cloning techniques (Sambrook and Russell, 2001).

### Generation of gene replacement cassettes

To express fluorescently labeled transcription factors XYR1 and CRE1 from their native loci, we choose a simultaneous knock-out/knock-in strategy. For this, gene replacements cassettes were constructed, that exchanged the complete *xyr1* or *cre1* open reading frame with full-length copies of each *gfp*-fusion gene plus a hygromycin resistance marker; the latter expressing the *E. coli* hygromycin B phosphotransferase gene (*hph*) under control of the promoter and terminator region of *T. reesei* glyceraldehyde 3-phosphate dehydrogenase (*gpd1*) gene (Hartl., 2007). Homologous recombination was facilitated via the native ∼ 1 kb 5′ and 3′ non-coding regions (Fig.S1). Assembly of the *xyr1* and *cre1* gene replacement cassettes was performed using InFusion® recombinational PCR cloning (cat.no. 639649, Takara Bio Europe/Clontech, Saint-Germain-en-Laye, France) with oligonucleotides summarized in Table S3. Briefly, pLH1-hph (Hartl., 2007) was first double-digested with XhoI/SalI and the linearized 5 kb vector backbone was purified by gel extraction. Subsequently, the 1 kb non-coding 3′-flanks of *xyr1* and *cre1* were PCR-amplified from genomic *T. reesei* DNA and inserted into pLH1-hph to generate pLH1-hph-X3f and pLH1-hph-C3f respectively. Next, pLH1-hph-X3f and pLH1-hph-C3f were reopened by BamHI restriction digestion, gel-purified, and the ORFs of *xyr1* (4.8 kb) and *cre1* (3.1 kb), each comprising 1 kb of the promoter region, the gene coding sequence, and 850bp of the terminator region, were amplified from *T. reesei* genomic DNA and integrated into the linearized vector backbone to result in pXYR1 and pCRE1. Finally, pXYR1 and pCRE1 were converted by full-length PCR amplification into linear DNA fragments of 11 and 9.1 kb length, respectively, and the GFP-coding sequences (750 bp), beforehand amplified from pAL1-MAK1 (Lichius., 2012), were joined in to generate pGFP–XYR1, pXYR1–GFP, pGFP–CRE1 and pCRE1–GFP. All generated plasmids were verified by control restriction digestion and sequencing. Correct plasmids were amplified through *E. coli*, and subsequently used as templates for the amplification of the 9.5 and 7.6 kb sized *xyr1* and *cre1* replacement cassettes, respectively, which were transformed into *T. reesei*.

### Transformation of *T. reesei*

Gene replacements cassettes were amplified from the respective plasmids by PCR and transformed into *T. reesei* QM 9414 *Δtku70* as linear DNA fragments using electroporation as described previously (Schuster., 2012). Transformants were selected on PDA hygromycin medium (100 μg ml^−1^ final concentration) Homokaryons were obtained by repeated rounds of vegetative spore propagation on selective medium and individual isolates were genotypically verified to confirm targeted integration of the replacement cassette by PCR as described in detail elsewhere (Lichius., 2012), using genotyping primers listed in Table S3.

### Real-time monitoring of transcription factor shuttling

In order to monitor rapid changes in the subcellular localization of fluorescently labeled XYR1 and CRE1 upon cellulase induction or repression, respectively, carbon source replacement experiments were performed using submerged germling cultures. For this, conidia from 1-week-old carbon source-free MA plate cultures were harvested in sterile water and cell concentration was determined using a Thoma cell counting chamber. A total of 1 × 10^8^ cells were used to inoculate 100 ml MA pre-culture medium (liquid MA medium with peptone to aid germination) in 500 ml shake flasks, supplemented with either 1% w/v cellulose in its carboxymethyl form (cellulase inducing condition) or 1% w/v glucose (cellulase repressing condition) as sole carbon source, and incubated at 28°C and 200 r.p.m. overnight in the dark. The next morning biomass from 20 ml pre-culture aliquots were washed twice with sterile tap water and transferred into 20 ml carbon source-free MA replacement medium (liquid MA medium without peptone) in 100 ml shake flasks. Initially, germlings were starved for up to 1.5 h under identical incubation conditions to deplete cell internal carbon storage. This, however, was not generally required and thus omitted during later experiments. At time point t = 0 min a new carbon source for either cellulase and xylanase induction [0.062% w/v sophorose (1.4 mM), 0.015% w/v xylose (1 mM), 1% w/v lactose (25 mM) or 1% w/v cellulose] or for cellulase repression [1% w/v glucose (50 mM) or 1% w/v xylose (65 mM)] was added and incubation continued. Germling samples for microscopical analysis were drawn from overnight pre-cultures as well as before and after carbon source replacement at desired time points. Carbon source-free MA cultures were used as controls for de-repressing conditions.

### Transcription factor recruitment colony profiling

In order to quantify localized nuclear recruitment of fluorescently labeled XYR1 and CRE1 in the three main regions of the functionally stratified fungal colony (periphery/leading edge, subperiphery and central area) MA agar plates supplemented with the respective carbon source for cellulase-inducing and -repressing conditions were centrally inoculated with *T. reesei* strains expressing either GFP–XYR1 or CRE1–GFP. Depending on the rate of colony development on the various carbon sources (growth on complex carbon sources generally required longer incubation times than growth on monosaccharides), incubation was performed for 48–72 h at 28°C and 12 h/12 h light/dark cycles until all three functional zones have been established and conidiation commenced. Agar block samples carrying mycelial sectors representative for all three main colony regions were cut out and prepared for live-cell imaging analysis.

### Quantitative live-cell imaging

Expression and subcellular localization of GFP-labeled transcription factors was quantified using scanning confocal microscopy and image analysis. Fungal cells were prepared either as germling samples from submerged flask cultures, simply by sandwiching 20 μl of cell suspension between two glass coverslips, or as mycelial samples prepared from growing plate cultures using the ‘inverted agar block’ method (Hickey., 2005).

Live-cell imaging was performed using a Nikon C1 confocal laser scanning unit mounted on a Nikon Eclipse TE2000-E inverted microscope base (Nikon GmbH, Vienna, Austria). GFP-labeled proteins were excited with the 488 nm laser line of an argon ion laser, and emitted fluorescence light separated by a Nikon MHX40500b/C100332 filter cube was detected with a photomultiplier tube within the range of 500–530 nm. A Nikon Plan Apo VC 60×/1.2 water immersion objective lens was used, and laser intensity and laser dwell time during image acquisition were kept to a minimum to reduce photobleaching and phototoxic effects while providing a sufficient signal-to-noise ratio for quantitative image analysis. Bright-field images were captured simultaneously with a Nikon C1-TD transmitted light detector mounted behind the condenser turret. Images were recorded with a maximum resolution of 1024 × 1024 pixels and saved as TIFF. Apart from display range adjustments and cropping using the ImageJ software platform (http://rsb.info.nih.gov/ij/), images were not subjected to further manipulation. Fluorescence intensity measurements and interactive 3D surface plots were performed with the corresponding plugins of the MacBiophotonics ImageJ work package available at (https://www.macbiophotonics.ca/software.htm), and statistically evaluated using Microsoft Excel.

For each data bar, up to 12 images covering 160 × 160 μm field of view of mycelial sample from the respective colony areas or of 25 μl submerged germling cultures, were randomly recorded, and in those images mean fluorescence intensities of 120 nuclei and 120 surrounding cytoplasmic areas were measured and averaged.

*Trichoderma reesei* is a multinucleate organism, and its nuclei are not mitotically synchronized. Thus, to account for the inherently high degree of variation in gene regulation between hyphae and within the nuclear population of one hypha, we calculated the nucleo-cytoplasmic fluorescence ratio (n/c-ratio) for each tested condition (Fig.S1). This value represents the ratio between the average nuclear fluorescence intensity within the population of nuclei and the average cytoplasmic fluorescence intensity between these nuclei, and should compensate for local differences in transcription factor expression and subcellular localization within the imaged hyphal population. Taking the detection limit of the microscope into account, an n/c-ratio of ≥ 1.3 indicates significant nuclear accumulation of fluorescently labeled transcription factor above residual autofluorescence background. Note: ‘error bars’ in the respective graphs represent the considerable biological variation of transcription factor recruitment to individual nuclei within the population rather than a statistical error (see example in Fig.S1C).

### Total RNA extraction from fungal biomass

In order to correlate nuclear presence or absence of both transcription factors with changes in gene expression, fungal biomass for subsequent RNA extraction and RT-qPCR analysis was collected immediately after the respective live-cell imaging shuttling experiments. For this, *T. reesei* germlings were harvested from induced or repressed 20 ml submerged cultures, and preserved by immediate shock-freezing in liquid nitrogen, followed by storage at −80°C. Total RNA extraction was performed according to Chirgwin. (1979), and RNA quality and quantity were determined using a NanoDrop spectrophotometer (Thermo Scientific, Vienna, Austria).

### Gene expression analysis by reverse transcriptase quantitative PCR (RT-qPCR)

DNase I-treated (cat.no. EN0521, Fermentas) RNA (3 μg) was reverse-transcribed with the RevertAid First Strand cDNA Kit (cat.no. K1632, Thermo Scientific) according to the manufacturer's protocol with a 1:1 combination of the provided oligo-dT and random hexamer primers. All RT-qPCR experiments were performed on an Eppendorf realplex^2^ Mastercycler (Eppendorf, Hamburg, Germany). Each sample was prepared as 25 μl reaction using the iQ SYBR Green Supermix (cat.no. 170-8882, Bio-Rad) with a final primer concentration of 100 nM forward and reverse primer each. All assays were carried out as triplicates in a 96-well plate format covered with optical tape, including no-template controls. Measurements with the housekeeping gene transcription elongation factor 1α (*tef1*) were performed for reference calculation and data normalization. Determination of the PCR efficiency was performed using triplicate reactions from a dilution series of cDNA (1; 0.1; 0.01; 0.001). Primers, amplification efficiency and R^2^ values are given in Table S2. Amplification efficiency of each sample mRNA was then calculated from the given slopes in the iQ5 Optical System Software v2.0 and relative fold-changes in gene expression were calculated using the Relative Expression Software Tool (REST) (http://www.gene-quantification.de/rest.html; Pfaffl., 2002). All samples were analyzed in two independent experiments with three replicates in each run.

### Protein extraction, SDS-PAGE and Western blot analysis

Overnight liquid cultures (100 ml MA medium, 1% w/v glucose) were prepared as described above for shuttling experiments. Samples for positive and negative controls, and for CRE1–GFP were directly drawn from these pre-cultures after 18 h of incubation. Biosynthesis of GFP–XYR1 was induced by transferring fungal biomass from 50 ml pre-culture into 50 ml fresh MA medium containing 1.4 mM sophorose. These induced cultures were harvested after 1, 2 and 3 h of additional incubation. For each sample, fungal biomass from 50 ml liquid pre- or induced culture was harvested on a glass microfiber filter (Whatmann, cat.no. 1822-047) using vacuum-driven filtration, and, after washing twice with sterile tap water, immediately shock frozen in liquid nitrogen. Subsequently, the biomass was ground to a fine powder in liquid nitrogen, and approximately 100 mg of it were added to a 2 ml Eppendorf tube already containing 1 ml of protein extraction buffer (10 ml PBS containing 5 mM EDTA and 5 mM PMSF plus one cOmplete ULTRA protease inhibitor cocktail tablet (Roche, cat.no. 05 892 791 001), pH 7.4), and 1 g of small (0.25 mm diameter) and four large (3 mm diameter) glass beads to aid cell destruction. The mix was subjected to three 1 min rounds of homogenization at 30 Hz with 1 min cooling intervals at −20°C. Cell debris and aqueous phase were separated by centrifugation with 17 000 *g* for 5 min and at 4°C. The clear supernatant containing all soluble proteins was transferred into fresh, pre-cooled Eppendorf tubes and stored at −20°C until further use. Total protein concentration was determined against BSA using Bradford reagent (Bio-Rad, cat.no. 500-0006) according to manufactures recommendations. Typically, protein yields between 1 and 3 mg ml^−1^ were achieved.

Except for the GFP control (cytosolic GFP expressed under control of the constitutive P*_tef1_* promoter in *T. reesei* QM6a) of which only 0.35 μg per lane were loaded, 20 μg of the crude total protein extract of all other samples were separated by SDS-PAGE as outlined in detail elsewhere (Sambrook and Russell, 2001). Generally, two identical 12% SDS-PAGE gels were prepared, one for colloidal Coomassie staining, and the second for semi-dry electro blotting of the separated proteins onto ImmobileFL PVDF membrane (Millipore, cat.no. IPFL00010). Subsequent blocking was achieved by incubation in PBS-T (PBS, 0.3% Tween 20) supplemented with 2% w/v milk powder (Roth, cat.no. T145.1) for 1 h at room temperature. For the specific labeling of GFP and GFP-fusion proteins, respectively, the monoclonal mouse anti-GFP–HRP antibody α-GFP(B-2) (Santa Cruz, cat.no. sc-9996) was used, diluted 1:1000 in PBS-T containing 0.5% w/v milk powder, and incubated on the membrane for 2 h at room temperature, followed by four washing steps with PBS-T.

Detection of the labeled proteins was performed with the Pierce ECL2 kit (Thermo Scientific, cat.no. 80197) according to manufactures recommendations. Chemifluorescent signals were recorded on a Typhoon FLA700 imager (GE Healthcare), and chemiluminescent signals were visualized by x-ray film (Amersham Hyperfilm ECL, GE Healthcare, cat.no. 28-9068-35) exposure. For protein band size estimation, two molecular weight markers were used: PageRuler Pre-stained Protein Ladder 10–170 kDa (Thermo Scientific, cat.no. 26616), and SuperSignal Enhanced Protein Ladder 20–150 kDa (Thermo Scientific, cat.no. 84786), with only the latter one being applicable for ECL detection on x-ray film.

## References

[b1] Akel E, Metz B, Seiboth B, Kubicek CP (2009). Molecular regulation of arabinan and L-arabinose metabolism in *Hypocrea jecorina**Trichoderma reesei*. Eukaryot Cell.

[b2] Arst HN, Tollervey D, Dowzer CE, Kelly JM (1990). An inversion truncating the *creA* gene of *Aspergillus nidulans* results in carbon catabolite derepression. Mol Microbiol.

[b3] Axelrod JD, Majors J, Brandriss MC (1991). Proline-independent binding of PUT3 transcriptional activator protein detected by footprinting *in vivo*. Mol Cell Biol.

[b4] Berger H, Pachlinger R, Morozov I, Goller S, Narendja F, Caddick M, Strauss J (2006). The GATA factor AreA regulates localization and *in vivo* binding site occupancy of the nitrate activator NirA. Mol Microbiol.

[b5] Bischof R, Fourtis L, Gamauf C, Seiboth B, Kubicek CP (2013). Comparative analysis of the *Trichoderma reesei* transcriptome during growth on the cellulase inducing substrates wheat straw and lactose. Biotechnol Biofuels.

[b6] Braun BR, van Het Hoog M, d'Enfert C, Martchenko M, Dungan J, Kuo A (2005). A human-curated annotation of the *Candida albicans* genome. PLoS Genet.

[b7] Calero-Nieto F, Di Pietro A, Roncero MI, Hera C (2007). Role of the transcriptional activator *xlnR* of *Fusarium oxysporum* in regulation of xylanase genes and virulence. Mol Plant Microbe Interact.

[b8] Chirgwin JM, Przybyla AE, MacDonald RJ, Rutter WJ (1979). Isolation of biologically active ribonucleic acid from sources enriched in ribonuclease. Biochemistry.

[b9] Chundawat SP, Beckham GT, Himmel ME, Dale BE (2011). Deconstruction of lignocellulosic biomass to fuels and chemicals. Ann Rev Chem Biomol Eng.

[b10] Collins GA, Tansey WP (2006). The proteasome: a utility tool for transcription?. Curr Opin Genet Dev.

[b11] Cziferszky A, Mach RL, Kubicek CP (2002). Phosphorylation positively regulates DNA binding of the carbon catabolite repressor Cre1 of *Hypocrea jecorina**Trichoderma reesei*. J Biolog Chem.

[b12] De Vit MJ, Waddle JA, Johnston M (1997). Regulated nuclear translocation of the Mig1 glucose repressor. Mol Biol Cell.

[b13] Deichsel H, Friedel S, Detterbeck A, Coyne C, Hamker U, MacWilliams HK (1999). Green fluorescent proteins with short half-lives as reporters in *Dictyostelium discoideum*. Dev Genes Evol.

[b14] DeVit MJ, Johnston M (1999). The nuclear exportin Msn5 is required for nuclear export of the Mig1 glucose repressor of *Saccharomyces cerevisiae*. Curr Biol.

[b15] Dinamarco TM, Almeida RS, de Castro PA, Brown NA, dos Reis TF, Ramalho LN (2012). Molecular characterization of the putative transcription factor SebA involved in virulence in *Aspergillus fumigatus*. Eukaryot Cell.

[b16] El Alami M, Feller A, Pierard A, Dubois E (2000). Characterisation of a tripartite nuclear localisation sequence in the regulatory protein Lys14 of *Saccharomyces cerevisiae*. Curr Genet.

[b17] Evdokimov AG, Pokross ME, Egorov NS, Zaraisky AG, Yampolsky IV, Merzlyak EM (2006). Structural basis for the fast maturation of *Arthropoda* green fluorescent protein. EMBO Rep.

[b18] Felenbok B, Flipphi M, Nikolaev I (2001). Ethanol catabolism in *Aspergillus nidulans*: a model system for studying gene regulation. Prog Nucleic Acid Res Mol Biol.

[b19] Foreman PK, Brown D, Dankmeyer L, Dean R, Diener S, Dunn-Coleman NS (2003). Transcriptional regulation of biomass-degrading enzymes in the filamentous fungus *Trichoderma reesei*. J Biol Chem.

[b20] Guangtao Z, Hartl L, Schuster A, Polak S, Schmoll M, Wang T (2009). Gene targeting in a nonhomologous end joining deficient *Hypocrea jecorina*. J Biotechnol.

[b21] Harbison CT, Gordon DB, Lee TI, Rinaldi NJ, Macisaac KD, Danford TW (2004). Transcriptional regulatory code of a eukaryotic genome. Nature.

[b22] Hartl L, Kubicek CP, Seiboth B (2007). Induction of the gal pathway and cellulase genes involves no transcriptional inducer function of the galactokinase in *Hypocrea jecorina*. J Biol Chem.

[b23] Hasper AA, Visser J, de Graaff LH (2000). The *Aspergillus niger* transcriptional activator XlnR, which is involved in the degradation of the polysaccharides xylan and cellulose, also regulates D-xylose reductase gene expression. Mol Microbiol.

[b24] Hasper AA, Trindade LM, van der Veen D, van Ooyen AJ, de Graaff LH (2004). Functional analysis of the transcriptional activator XlnR from *Aspergillus niger*. Microbiology.

[b25] Hayakawa Y, Ishikawa E, Shoji JY, Nakano H, Kitamoto K (2011). Septum-directed secretion in the filamentous fungus *Aspergillus oryzae*. Mol Microbiol.

[b26] Häkkinen M, Valkonen MJ, Westerholm-Parvinen A, Aro N, Arvas M, Vitikainen M (2014). Screening of candidate regulators for cellulase and hemicellulase production in *Trichoderma reesei* and identification of a factor essential for cellulase production. Biotechnol Biofuels.

[b27] Heim R, Tsien RY (1996). Engineering green fluorescent protein for improved brightness, longer wavelengths and fluorescence resonance energy transfer. Curr Biol.

[b28] Heim R, Cubitt AB, Tsien RY (1995). Improved green fluorescence. Nature.

[b29] Herold S, Bischof R, Metz B, Seiboth B, Kubicek CP (2013). Xylanase gene transcription in *Trichoderma reesei* is triggered by different inducers representing different hemicellulosic pentose polymers. Eukaryot Cell.

[b30] Hickey PC, Swift SR, Roca MG, Charalabos P, Read ND, Savidge T (2005). Live-cell Imaging of filamentous fungi using vital fluorescent dyes and confocal microscopy. Methods in Microbiology.

[b31] Ilmen M, Thrane C, Penttila M (1996). The glucose repressor gene *cre1* of *Trichoderma*: isolation and expression of a full-length and a truncated mutant form. Mol Gen Genet.

[b32] Jekosch K, Kück U (2000). Loss of glucose repression in an *Acremonium chrysogenum* beta-lactam producer strain and its restoration by multiple copies of the *cre1* gene. Appl Microbiol Biotechnol.

[b33] Kirkpatrick CR, Schimmel P (1995). Detection of leucine-independent DNA site occupancy of the yeast Leu3p transcriptional activator *in vivo*. Mol Cell Biol.

[b34] Klaubauf S, Narang HM, Post H, Zhou M, Brunner K, Mach-Aigner AR (2014). Similar is not the same: differences in the function of the (hemi-)cellulolytic regulator XlnR (Xlr1/Xyr1) in filamentous fungi. Fungal Genet Biol.

[b35] Kohlhaw GB (2003). Leucine biosynthesis in fungi: entering metabolism through the back door. Microbiol Mol Biol Rev.

[b36] Kren A, Mamnun YM, Bauer BE, Schuller C, Wolfger H, Hatzixanthis K (2003). War1p, a novel transcription factor controlling weak acid stress response in yeast. Mol Cell Biol.

[b37] Kubicek CP (2013). Systems biological approaches towards understanding cellulase production by *Trichoderma reesei*. J Biotechnol.

[b38] Kubicek CP, Mikus M, Schuster A, Schmoll M, Seiboth B (2009). Metabolic engineering strategies for the improvement of cellulase production by *Hypocrea jecorina*. Biotechnol Biofuels.

[b39] Kubodera T, Yamashita N, Nishimura A (2002). Transformation of *Aspergillus* sp. and *Trichoderma reesei* using the pyrithiamine resistance gene (*ptrA*) of *Aspergillus oryzae*. Biosci Biotechnol Biochem.

[b40] Levin AM, de Vries RP, Conesa A, de Bekker C, Talon M, Menke HH (2007a). Spatial differentiation in the vegetative mycelium of *Aspergillus niger*. Eukaryot Cell.

[b41] Levin AM, de Vries RP, Wosten HA (2007b). Localization of protein secretion in fungal colonies using a novel culturing technique; the ring-plate system. J Microbiol Methods.

[b42] Lichius A, Lord KM, Jeffree CE, Oborny R, Boonyarungsrit P, Read ND (2012). Importance of MAP kinases during protoperithecial morphogenesis in *Neurospora crassa*. PLoS ONE.

[b43] Mach RL, Strauss J, Zeilinger S, Schindler M, Kubicek CP (1996). Carbon catabolite repression of xylanase I (*xyn1*) gene expression in *Trichoderma reesei*. Mol Microbiol.

[b44] Mach-Aigner AR, Pucher ME, Steiger MG, Bauer GE, Preis SJ, Mach RL (2008). Transcriptional regulation of *xyr1*, encoding the main regulator of the xylanolytic and cellulolytic enzyme system in *Hypocrea jecorina*. Appl Environ Microbiol.

[b45] MacPherson S, Larochelle M, Turcotte B (2006). A fungal family of transcriptional regulators: the zinc cluster proteins. Microbiol Mol Biol Rev.

[b46] Makita T, Katsuyama Y, Tani S, Suzuki H, Kato N, Todd RB (2009). Inducer-dependent nuclear localization of a Zn(II)(2)Cys(6) transcriptional activator, AmyR, in *Aspergillus nidulans*. Biosci Biotechnol Biochem.

[b47] Mandels MM, Andreotti RE (1978). The cellulose to cellulase fermentation. Proc Biochem.

[b48] Marui J, Tanaka A, Mimura S, de Graaff LH, Visser J, Kitamoto N (2002). A transcriptional activator, AoXlnR, controls the expression of genes encoding xylanolytic enzymes in *Aspergillus oryzae*. Fungal Genet Biol.

[b49] Metz B, Seidl-Seiboth V, Haarmann T, Kopchinskiy A, Lorenz P, Seiboth B, Kubicek CP (2011). Expression of biomass-degrading enzymes is a major event during conidium development in *Trichoderma reesei*. Eukaryot Cell.

[b50] Muratani M, Kung C, Shokat KM, Tansey WP (2005). The F box protein Dsg1/Mdm30 is a transcriptional coactivator that stimulates Gal4 turnover and cotranscriptional mRNA processing. Cell.

[b51] Nehlin JO, Ronne H (1990). Yeast MIG1 repressor is related to the mammalian early growth response and Wilms' tumour finger proteins. EMBO J.

[b52] van Peij NN, Gielkens MM, de Vries RP, Visser J, de Graaff LH (1998). The transcriptional activator XlnR regulates both xylanolytic and endoglucanase gene expression in *Aspergillus niger*. Appl Environ Microbiol.

[b53] Pfaffl MW, Horgan GW, Dempfle L (2002). Relative expression software tool (REST) for group-wise comparison and statistical analysis of relative expression results in real-time PCR. Nucleic Acids Res.

[b54] Portnoy T, Margeot A, Linke R, Atanasova L, Fekete E, Sandor E (2011a). The CRE1 carbon catabolite repressor of the fungus *Trichoderma reesei*: a master regulator of carbon assimilation. BMC Genomics.

[b55] Portnoy T, Margeot A, Seidl-Seiboth V, Le Crom S, Ben Chaabane F, Linke R (2011b). Differential regulation of the cellulase transcription factors XYR1, ACE2, and ACE1 in *Trichoderma reesei* strains producing high and low levels of cellulase. Eukaryot Cell.

[b56] Read ND (2011). Exocytosis and growth do not occur only at hyphal tips. Mol Microbiol.

[b57] Roper M, Ellison C, Taylor JW, Glass NL (2011). Nuclear and genome dynamics in multinucleate ascomycete fungi. Curr Biol.

[b58] Roy P, Lockington RA, Kelly JM (2008). CreA-mediated repression in *Aspergillus nidulans* does not require transcriptional auto-regulation, regulated intracellular localisation or degradation of CreA. Fungal Genet Biol.

[b59] Sambrook J, Russell DW (2001). Molecular Cloning: A Laboratory Manual.

[b60] Schneider-Poetsch T, Ju J, Eyler DE, Dang Y, Bhat S, Merrick WC (2010). Inhibition of eukaryotic translation elongation by cycloheximide and lactimidomycin. Nat Chem Biol.

[b61] Schuster A, Bruno KS, Collett JR, Baker SE, Seiboth B, Kubicek CP, Schmoll M (2012). A versatile toolkit for high throughput functional genomics with *Trichoderma reesei*. Biotechnol Biofuels.

[b62] Seiboth B, Gamauf C, Pail M, Hartl L, Kubicek CP (2007). The D-xylose reductase of *Hypocrea jecorina* is the major aldose reductase in pentose and D-galactose catabolism and necessary for beta-galactosidase and cellulase induction by lactose. Mol Microbiol.

[b63] Seiboth B, Herold S, Kubicek CP (2012). Metabolic engineering of inducer formation for cellulase and hemicellulase gene expression in *Trichoderma reesei*. Subcell Biochem.

[b64] Sellick CA, Reece RJ (2003). Modulation of transcription factor function by an amino acid: activation of Put3p by proline. EMBO J.

[b65] Sellick CA, Reece RJ (2005). Eukaryotic transcription factors as direct nutrient sensors. Trends Biochem Sci.

[b66] Shimizu K, Hicks JK, Huang TP, Keller NP (2003). Pka, Ras and RGS protein interactions regulate activity of AflR, a Zn(II)2Cys6 transcription factor in *Aspergillus nidulans*. Genetics.

[b67] Shroff RA, Lockington RA, Kelly JM (1996). Analysis of mutations in the *creA* gene involved in carbon catabolite repression in *Aspergillus nidulans*. Can J Microbiol.

[b68] Strauss J, Mach RL, Zeilinger S, Hartler G, Stoffler G, Wolschek M, Kubicek CP (1995). Cre1, the carbon catabolite repressor protein from *Trichoderma reesei*. FEBS Lett.

[b69] Stricker AR, Grosstessner-Hain K, Wurleitner E, Mach RL (2006). Xyr1 (xylanase regulator 1) regulates both the hydrolytic enzyme system and D-xylose metabolism in *Hypocrea jecorina*. Eukaryot Cell.

[b70] Stricker AR, Mach RL, de Graaff LH (2008). Regulation of transcription of cellulases- and hemicellulases-encoding genes in *Aspergillus niger* and *Hypocrea jecorina**Trichoderma reesei*. Appl Microbiol Biotechnol.

[b71] Sun J, Tian C, Diamond S, Glass NL (2012). Deciphering transcriptional regulatory mechanisms associated with hemicellulose degradation in *Neurospora crassa*. Eukaryot Cell.

[b72] Tonukari NJ, Scott-Craig JS, Walton JD (2000). The *Cochliobolus carbonum* SNF1 gene is required for cell wall-degrading enzyme expression and virulence on maize. Plant Cell.

[b73] Vautard-Mey G, Cotton P, Fevre M (1999). Expression and compartmentation of the glucose repressor CRE1 from the phytopathogenic fungus *Sclerotinia sclerotiorum*. Eur J Biochem.

[b74] Viikari L, Vehmaanperä J, Koivula A (2012). Lignocellulosic ethanol: from science to industry. Biomass Bioenergy.

[b75] de Vries RP, Visser J, de Graaff LH (1999). CreA modulates the XlnR-induced expression on xylose of *Aspergillus niger* genes involved in xylan degradation. Res Microbiol.

[b76] Wightman R, Bell R, Reece RJ (2008). Localization and interaction of the proteins constituting the GAL genetic switch in *Saccharomyces cerevisiae*. Eukaryot Cell.

[b77] Yi M, Park JH, Ahn JH, Lee YH (2008). MoSNF1 regulates sporulation and pathogenicity in the rice blast fungus *Magnaporthe oryzae*. Fungal Genet Biol.

[b78] Zeilinger S, Mach RL, Schindler M, Herzog P, Kubicek CP (1996). Different inducibility of expression of the two xylanase genes *xyn1* and *xyn2* in *Trichoderma reesei*. J Biol Chem.

[b79] Zhao X, Duong T, Huang CC, Kain SR, Li X (1999). Comparison of enhanced green fluorescent protein and its destabilized form as transcription reporters. Methods Enzymol.

