# Supplementary material for: Nucleo-cytoplasmic shuttling dynamics of the transcriptional regulators XYR1 and CRE1 under conditions of cellulase and xylanase gene expression in Trichoderma reesei
Source: Mol Microbiol. 2014 Oct 29;94(5):1162–78. doi: 10.1111/mmi.12824 (PMC4282317; doi:10.1111/mmi.12824)
Supplement: Supplementary file 1 — Supporting information [file mmi0094-1162-sd1.zip › mmi12824-sup-0007-ts1-3.pdf]

SUPPLEMENTARY MATERIAL

Figure S1. *GFP-labeling of XYR1 and CRE1 at their native loci.* **(A)** Replacement cassettes were cloned for the expression of N- and C-terminally labeled XYR1 and CRE1 GFP-fusion proteins from their native loci. **(B)** All four GFP-fusions (XYR1-GFP, GFP-XYR1, CRE1-GFP and GFP-CRE1) localized to nuclei which was confirmed by co-staining with DAPI. Scale bar, 5 µm. **(C)** To analyze relative changes in subcellular transcription factor localization in response to different carbon sources, the nucleo/cytoplasmic fluorescence ratio (n/c-ratio) was determined by quantitative image analysis. For this, mean fluorescence intensities within 0.34 μm^2 circular areas - covering the average projection area of a single nucleus - were measured in at least 120 nuclei (full circles) and 120 adjacent cytoplasmic regions (broken circles) for each tested condition, and correlated. The indicated mean values demonstrate the considerable biological variation of nuclear fluorescence amongst the population of nuclei within individual hyphae (mean fluorescence intensity = 1002 ± 598 units). Scale bar, 2 µm. **(D)** Quantification of nuclear import efficiency of N- and C-terminally labeled GFP-fusion constructs upon cellulase induction (lactose) for XYR1 and repression (glucose) for CRE1, revealed that GFP-XYR1 and CRE1-GFP constitute the fully functional fusion constructs.

Figure S2. *Growth analysis of xyr1 and cre1 transformants.* **(A)** Colony extension measurements on various carbon sources. Notably, differences in the average colony extension rate affected by defects in *xyr1* expression or XYR1 function, occurred exclusively on high concentrations of xylose [65mM] as sole carbon source. Expression of GFP-XYR1 in QM9414 Δ*tku70* transformants (clones GX1 and GX2; green arrowhead) showed fast colony

This article is protected by copyright. All rights reserved.
This is an open access article under the terms of the Creative Commons Attribution License, which permits use, distribution and reproduction in any medium, provided the original work is properly cited.

extension rates comparable to the parental strain, whereas expression of XYR1-GFP (clones XG1 and XG2; red arrowhead) only partially compensated the growth defect typical for Δ*xyr1* (black arrowhead) under these conditions. Similarly, only the CRE1-GFP fusion construct (clones CG1 and CG2; blue arrowhead) allowed wild-type like colony development, whereas expression of GFP-CRE1 (clones GC1 and GC2) lead to a similar decrease in average colony extension rate as seen in XG1 and XG2. Colony development of Δ*cre1* was severely impaired independently of the used carbon source (asterisks). **(B)** Colony development is influenced by the available carbon source. On glucose and xylose (cellulase repression) dense vegetative mycelia with late onset of conidiation developed, whereas on sophorose and cellulose (cellulase induction) vegetative mycelia developed much sparser but with more vigorous and early onset conidiation. On lactose, an intermediate morphology developed. All cultures have been incubated for 72h at 28°C with 12h/12h light/dark-cycles on MA medium with the indicated carbon sources.

Figure S3. *Subcellular localization of XYR1 and CRE1 in response to different inducing and non-inducing carbon sources.* Overnight submerged germling pre-cultures cultivated on glycerol as carbon source, were replaced into carbon-free medium and starved for 1.5 h to remove cell-internal carbon storage. Upon addition of the new carbon source, changes in the subcellular localization of fluorescently-labelled transcription factors were measured by quantitative live-cell imaging at indicated time points. **(A)** Nuclear import of XYR1 was rapidly and most effectively triggered with sophorose (n/c-ratio increase to 6.6). Cellulose (CMC) and lactose required extended incubation times (> 19 h) to result noticeable increase in nuclear fluorescence. Non-inducing carbon sources (xylose, glucose and glycerol) did not

This article is protected by copyright. All rights reserved.
This is an open access article under the terms of the Creative Commons Attribution License, which permits use, distribution and reproduction in any medium, provided the original work is properly cited.

trigger significant nuclear import of XYR1 above pre-culture control level. **(B)** Nuclear import of CRE1 was most efficiently induced with glucose (n/c-ratio increase to 6.6), followed by xylose and glycerol. Lactose and cellulose showed weak effects but only after extended incubation times. All carbon sources were used at 1% w/v, except for sophorose (0.06% w/v 0 1.4mM).

Figure S4. *Nuclei of T. reesei stain negatively against a GFP-filled cytoplasm.* Three consecutive Z-stack images of a *T. reesei* strain constitutively expressing GFP in the cytoplasm in which nuclei have been stained with the DNA dye DAPI. Nuclei appear as dark, non-fluorescent dots in the brightly fluorescent cytoplasm, suggesting that GFP freely expressed in the cytoplasm does not readily cross through nuclear pore complexes. Cytoplasmic streaming and technical limitations of the confocal microscope (sequential imaging of DAPI and GFP signals and slow line scanning image acquisition) do not allow perfect alignment of DAPI-stained nuclei with their negatively stained positions in the cytoplasm in the merge images. Scale bars, 5 µm.

Figure S5. *Comparison of XYR1 nuclear recruitment and gene expression upon glucose-to-sophorose replacement and sophorose addition to glucose pre-culture.* No significant differences in XYR1 import dynamics and subsequent marker gene expression were observed whether sophorose was added with our without replacement of the glucose containing pre-culture medium. This demonstrates that residual amounts of glucose do not interfere with cellulase induction by 1.4 mM sophorose.

This article is protected by copyright. All rights reserved.
This is an open access article under the terms of the Creative Commons Attribution License, which permits use, distribution and reproduction in any medium, provided the original work is properly cited.

Figure S6. *The cytoplasmic pool of XYR1 increased in the central area of the colony on sophorose***.** Nuclear recruitment of XYR1 within the three functional zones of the colony, quantified in two independent GFP-XYR1 transformants (clones GX1 and GX2) under cellulase inducing conditions. Notably, on sophorose the cytoplasmic signal of XYR1 is significantly elevated in comparison to the other three zones of the same colony, and in comparison to all zones of the same strains grown on lactose, leading to a low n/c-ratio. This might indicate that the main region of XYR1 synthesis is also located in the colony center. All strains were evaluated after 48h incubation on MA medium supplemented with the indicated carbon source.

This article is protected by copyright. All rights reserved.
This is an open access article under the terms of the Creative Commons Attribution License, which permits use, distribution and reproduction in any medium, provided the original work is properly cited.

Table S1. T.reesei *strains used and produced in this study.*

Strain	***Strain number***	***Parental Strain***	***reference***
QM9414	ATCC 26921	QM6a	(Mandels & Andreotti, 1978)
QM9414 Δ*tku70*	-	QM9414	C. Ivanova,, unpublished data
Δ*xyr1*	-	QM9414	(Stricker et al., 2006)
Δ*cre1*	-	QM9414	(Portnoy et al., 2011b)
GFP	-	QM6a, P_{tef1}::*gfp*	R. Linke, unpublished data
XYR1-GFP	TRAL001	QM9414 Δ*tku70*	this study
GFP-XYR1	TRAL002	QM9414 Δ*tku70*	this study
CRE1-GFP	TRAL003	QM9414	this study
GFP-CRE1	TRAL004	QM9414	this study

This article is protected by copyright. All rights reserved.
This is an open access article under the terms of the Creative Commons Attribution License, which permits use, distribution and reproduction in any medium, provided the original work is properly cited.

Table S2. *RT-qPCR oligonucleotides used in this study.*

Gene	*Primer name*	*5'-3' Sequence*	R^2	*Efficiency*
cbh1	qPCR-cbh1-F	CCGAGCTTGGTAGTTACTCTG	0.990	98%
	qPCR-cbh1-R	GGTAGCCTTCTTGAACTGAGT		
xyn2	qPCR-xyn2-F	CAACCAGCCGTCCATCATCG	0.993	97%
	qPCR-xyn2-R	ATCGTCCCGAGCGTCAGG		
xyr1	qPCR-xyr1-F	CCATCAACCTTCTAGACGAC	0.987	99%
	qPCR-xyr1-R	AACCCTGCAGGAGATAGAC		
cre1	qPCR-cre1-F	GTCTGAGAAACCTGTCCCTG	0.996	91%
	qPCR-cre1-R	GGCTAATGATGTCGGTAAGTG		
tef1	qPCR-tef1-F	CCACATTGCCTGCAAGTTCGC	0.995	95%
	qPCR-tef1-R	GTCGGTGAAAGCCTCAACGCA		

This article is protected by copyright. All rights reserved.
This is an open access article under the terms of the Creative Commons Attribution License, which permits use, distribution and reproduction in any medium, provided the original work is properly cited.

Table S3. *Oligonucleotides used in this study.* Compatible overhangs required for InFusion® recombinational cloning are indicated in bold, restriction enzyme recognition sites are underlined.

Primer name	***5'-3' Sequence***
Oligonicleotides for InFusion recombinational cloning	
xyr1-3'-flank-F	**CCATAGTACCCTCGAG**GCAACACAACACTCACCTCTT
xyr1-3'-flank-R	**ATGCCTGCAGGTCGACGCTAGC**AGAATAGGAGGATGGCTCTTG
Pxyr1-F	**CGGTACCCGGGGATCCGCTAGC**ACACAAGAGCAATGGCCCTAGC
Txyr1-R	**GGTAGCTCTCGGGATCC**ACTCGTCACACTGGCTCTCGTAC
N-term-GFP-F	**CAGCGCGCCACA**ATGGTGAGCAAGGGCGAGGA
N-term-GFP-R	**GCCTCCGCCTCCGCCTCCGCCGCCTCCGCC**CTTGTACAGCTCGTCCATGC
N-term-pXYR1-F	**GGCGGAGGCGGAGGC**TTGTCCAATCCTCTCCGTCGC
N-term-pXYR1-R	**GCCCTTGCTCACCAT**TGTGGCGCGCTGTGTGCGA
C-term-GFP-F	**GGCGGAGGCGGAGGC**GTGAGCAAGGGCGAGGAG
C-term-GFP-R	**GATTGAGTGGCCTCC**TTACTTGTACAGCTCGTCCATGC
C-term-pXYR1-F	**GAGCTGTACAAGTAA**GGAGGCCACTCAATCGTATG
C-term-pXYR1-R	**GCCTCCGCCTCCGCCTCCGCCGCCTCCGCC**GAGGGCCAGACCGGTTCCGT
cre1-3'-flank-F	**CCATAGTACCCTCGAG**CCAAGCCTTCTCCGACGTTTC
cre1-3'-flank-R	**ATGCCTGCAGGTCGACGCTAGC**AGCAATAGAGCCACAGTCAAGGTT
Pcre1-F	**CGGTACCCGGGGATCCGCTAGC**CGTGTCCAAGTACCCTCCGTC
Tcre1-R	**GGTAGCTCTCGGATCC**CCCGTGGTCAGTCCTCTCATC
N-term-GFP-F	**CTGCCGCTCGATCAC**ATGGTGAGCAAGGGCGAGGA
N-term-GFP-R	**GCCTCCGCCTCCGCCTCCGCCGCCTCCGCC**CTTGTACAGCTCGTCCATGC
N-term-pCRE1-F	**GGCGGAGGCGGAGGCC**AACGAGCACAGTCTGCCGT
N-term-pCRE1-R	**GCCCTTGCTCACCAT**GTGATCGAGCGGCAGTCAAA
C-term-GFP-F	**GGCGGAGGCGGAGGC**GTGAGCAAGGGCGAGGAG
C-term-GFP-R	**TGAGTACCGGACATT**TTACTTGTACAGCTCGTCCATGC
C-term-pCRE1-F	**GAGCTGTACAAGTAA**AATGTCCGGTACTCATGGCG
C-term-pCRE1-R	**GCCTCCGCCTCCGCCTCCGCCGCCTCCGCC**CATCCGATCCATGAGGTCGC
Oligonucleotides for gene replacement cassette amplification	
Pxyr1-F	ACACAAGAGCAATGGCCCTAGC
xyr1-3f-R	CAATCTCGATCCTGAGGGCTTC
Pcre1-F	CGTGTCCAAGTACCCTCCGTC
cre1-3f-R	CAGCAATAGAGCCACAGTCAAGG
Oligonucleotides for PCR-genotyping	

This article is protected by copyright. All rights reserved.
This is an open access article under the terms of the Creative Commons Attribution License, which permits use, distribution and reproduction in any medium, provided the original work is properly cited.

5Pxyr1-ver-F	CCAGCTGCCACTCTCATG
Xyr1-ver-R	CCTGGCAGCAATAAGAGAGC
Xyr1-ver-F	CCTTGCGGATAAGTGGGATC
5Pcre1-ver-F	TGAGTAGAAAAGAGGACACGGC
Cre1-ver-R	CGTGTGGGTGCGAATGT
Cre1-ver-F	CGTACCACATGGCGAGAG
GFP-ver-R	AAGCACTGCACGCCGTA
GFP-ver-F	TACGGCGTGCAGTGCTTC
Pgpd1-ver-F	TGCTAAGGTACCTAGGGAGGGA
hph-ver-R	CAAGCACTTCCGGAATCG
act-mp-F	ACTTTCGGCCGCATTCTG
act-mp-R	AGCCAGGATCTTCATCAGGTAG

This article is protected by copyright. All rights reserved.
This is an open access article under the terms of the Creative Commons Attribution License, which permits use, distribution and reproduction in any medium, provided the original work is properly cited.
